# Supplementary material for: Fluid network dynamics in the prefrontal cortex during multiple strategy switching
Source: Nat Commun. 2018 Jan 22;9:309. doi: 10.1038/s41467-017-02764-x (PMC5778086; doi:10.1038/s41467-017-02764-x)
Supplement: Supplementary file 1 — Supplementary Information [file 41467_2017_2764_MOESM1_ESM.pdf]

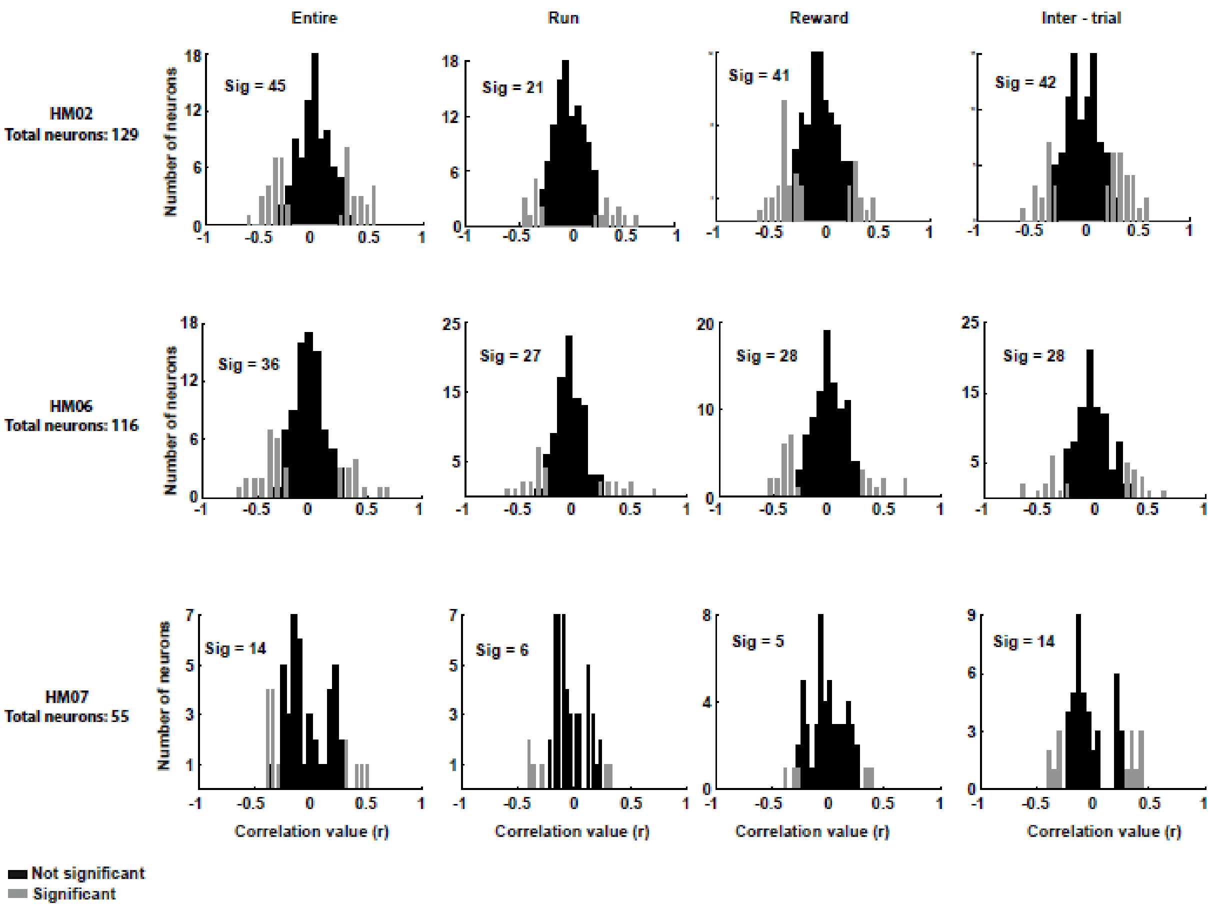

Supplementary figure 1 | Histograms of correlation values between firing rate and performance per animal. An entire trial can be subdivided into three different segments: The run segment starts when the animal initiates movement towards the goal arm, and it ends when the animal crosses the reward sensor located at the goal arm; The reward segment starts when the animal crosses the reward sensor and it lasts for two seconds; the Inter – Trial segment starts with the end of the reward segment and lasts till the beginning of the next run segment. It comprises the time that the animal spends in a bin between trials. Neuronal firing rates are calculated for each of these four periods (Entire, Run, Reward, and Inter-Trial) and are correlated to the animal performance. The figure shows the histograms of correlations between the firing rate of individual neurons, in each one of the four different segments, and the performance of the animal (300 neurons). The number of neurons with significantly correlated (grey bars) firing rates to task performance are denoted with 'Sig' (Spearman´s correlation, $p < 0.05$ after Bonferroni-Holm correction).

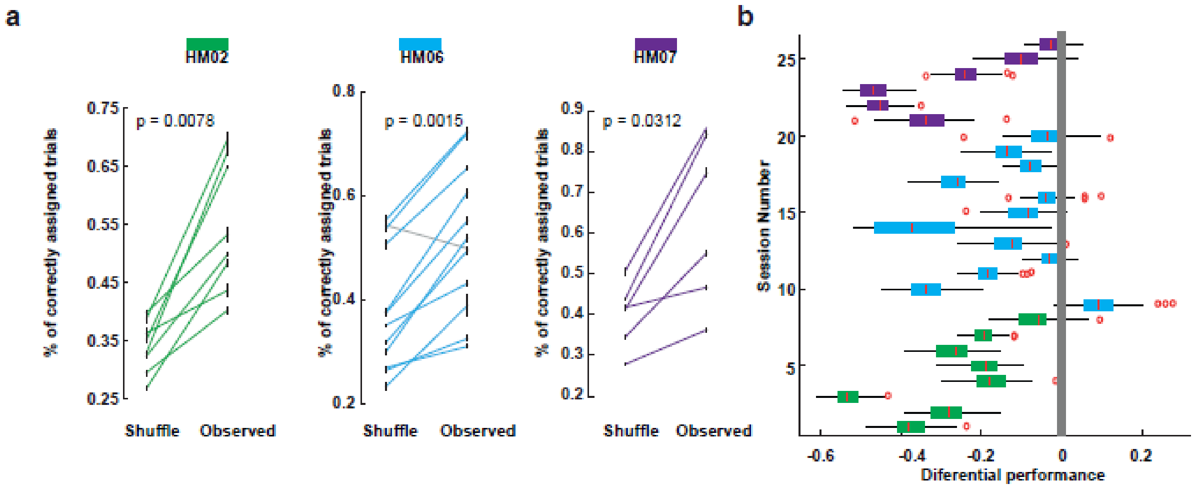

Supplementary figure 2 | K-means clustering analyses of neuronal state for each individual animal. (**a**) Same procedure as in Fig. 4c, after creating a neuronal state network in which each trial is represented by the individual firing rate of all the neurons during that trial, the k-means clustering algorithm was applied. K-means was used to cluster the trials of a recording session according to the rules presented during that session. After comparing the final assignation of trials, by the k-means, to the observed data, we created an accuracy percentage by dividing the number of correctly assigned trials over the total number of trials. For each neuron, we shuffled the trial-by-trial firing rates, resulting in a random representation of the neuronal state network. We applied a k-means algorithm and again computed an accuracy value for the shuffled data. Comparison between the performance of k-means clustering algorithm applied to the observed neuronal state network versus the performance of the k-means clustering applied to the shuffled neuronal state network is presented for each individual animal (p values were calculated using Wilcoxon signed rank test). (**b**) Same procedure as described in **a**, with the difference that the starting centroids of the k-means algorithm were defined as the observed rule centres in the recorded data. The accuracy of the clustering over the observed data is represented by the grey line, and accuracies of the k-means applied to the shuffled data are shown as the difference from the observed accuracy. Animals are colour coded (HM02 = green, HM06 = blue, HM07 = purple).

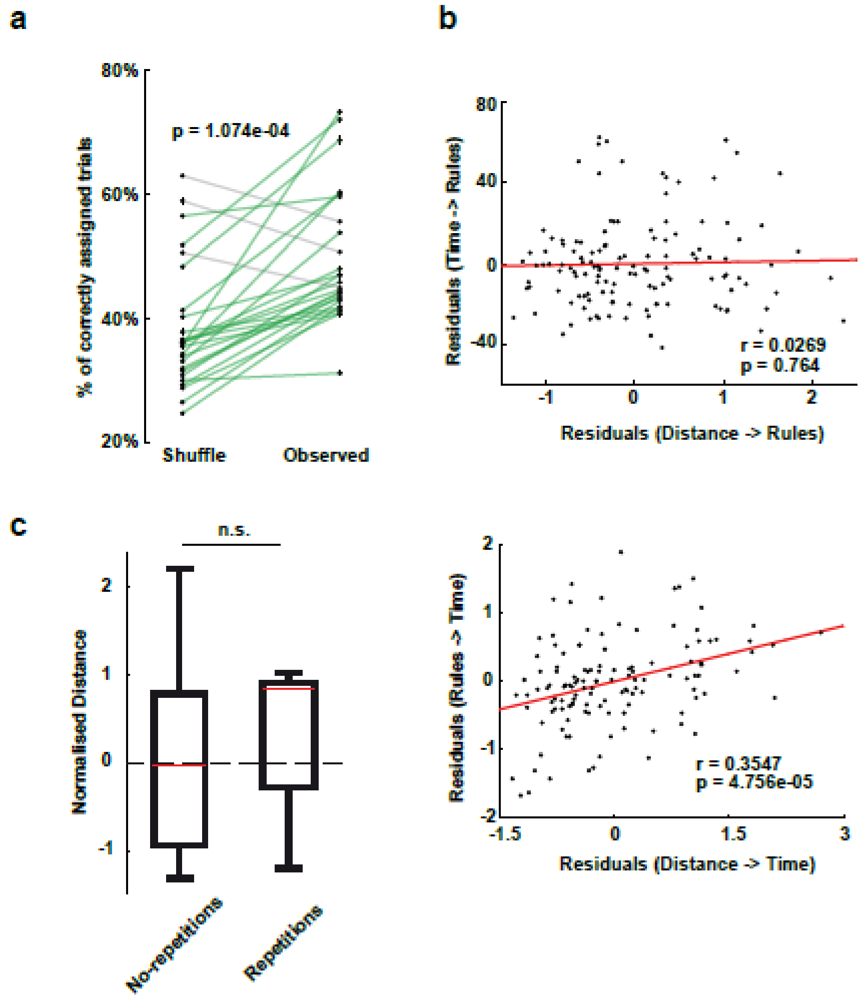

Supplementary figure 3 | Analyses of the neuronal state network using Mahalanobis distance. (**a**) Similar to Fig. 4c, after creating a neuronal state network in which each trial is represented by the individual firing rate of all the neurons in that trial, k-medoids clustering algorithm (similar to k-means but using Mahalanobis distance) was applied. K-medoids was used to cluster the trials of a recording session according to the rules presented during that session. After comparing the final assignation of trials by the k-medoids to the observed data, we created an accuracy percentage by dividing the number of correctly assigned trials over the total number. Then, for each neuron, we shuffled the trial-by-trial firing rates, resulting in a random representation of the trials in the neuronal state network. We applied the k-medoids algorithm and again computed an accuracy value for the shuffled data. K-medoids clustering algorithm applied to the observed neuronal state network is significantly better than the performance of the k-medoids clustering applied to the shuffled neuronal state network (p = 1.074e-04, Wilcoxon signed rank test). (**b**) Mahalanobis distance between rule clusters was calculated for each possible rule pair in a recording session. Any pair of rules within a session were described by three factors: the Mahalanobis distance between them; the number of additional rule clusters in between; and the number of the trials in between. Similar to Fig. 4f, two partial correlations are plotted. Top, the partial correlation between the

Mahalanobis distance between clusters and the number of trials in between, while taking into account the number or rule clusters in between ($p = 0.764$, $r = 0.0269$, Spearman correlation). Bottom, the partial correlation between the Mahalanobis distance between clusters and the number of rule clusters in between, taking into account the number of trials in between ($p = 4.756e-05$, $r = 0.3547$, Spearman correlation). (**c**) Box plot indicating the normalised distance (using Mahalanobis) measured between all possible pairs of rule clusters. The distance was normalised by subtracting the mean distance of all the distances between rule clusters with the same number of rules in between. Red line indicates the median. The normalised distances between a rule 'A' and its repetition 'A°' were not significantly different from all the other normalised distances between two different rules ($p = 0.24$, Wilcoxon rank sum test).

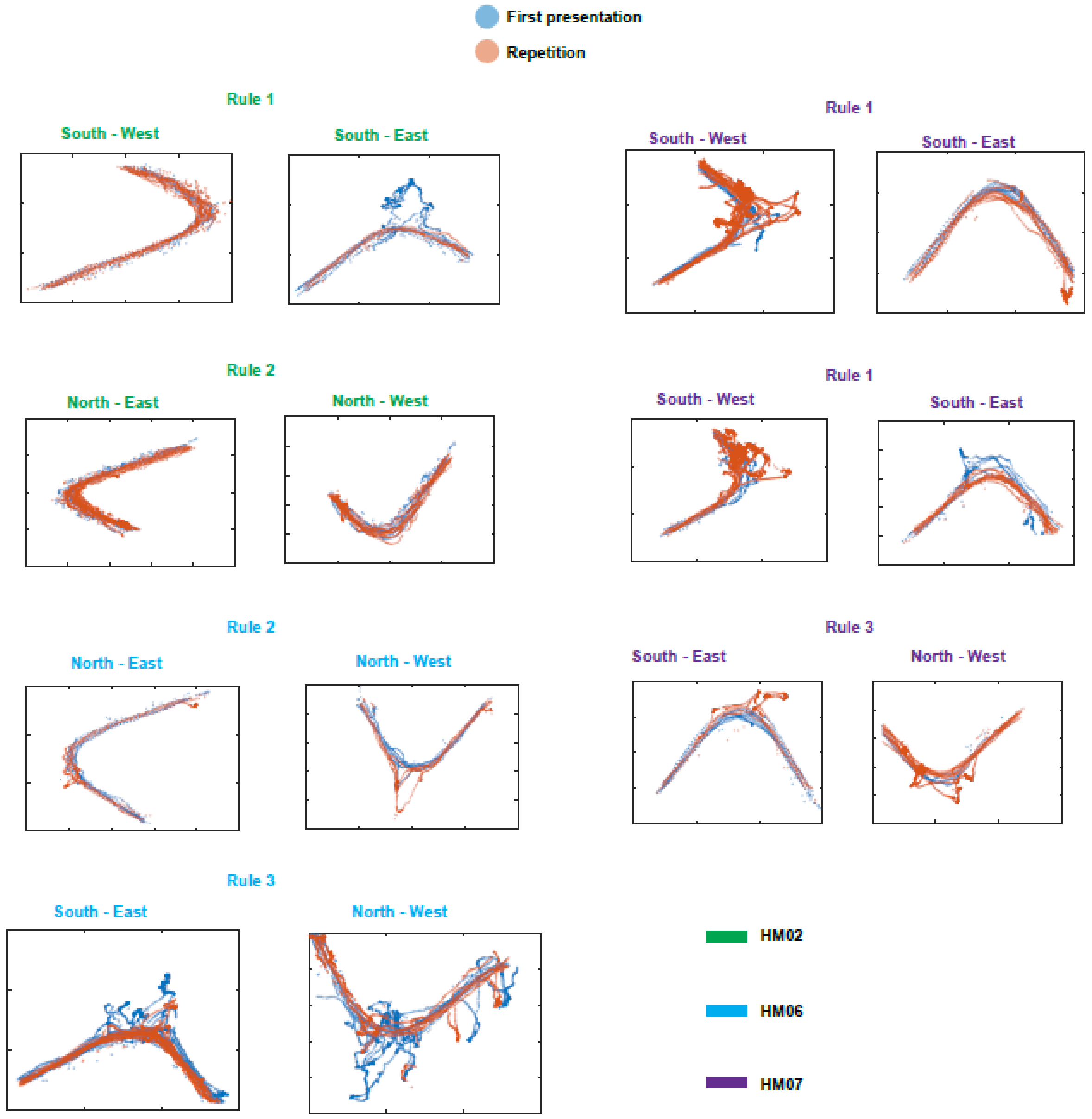

Supplementary figure 4 | Overlapping trajectories between a rule ‘A’ and its later repetition ‘A°’. Overlapping trajectories between a rule (blue dots) and its later repetition (red dots) during the same recording day. Individual animals are colour-coded.

Animal	p-value (# Rules)	p-value (Trials in between)
HM02	4.07e-05	0.593
HM06	0.003	0.753
HM07	0.097	0.99
All animals	2.81e-08	0.78

Supplementary Table 1: Individual animals´ p-values of the regressors (Number of rules and trials in between) explaining the distance between rule clusters. When having a pair of rule clusters in a given session, the distance between these clusters in the neuronal state network could be explained by either the number of rule clusters in between or the number of trials in between. After fitting a general linear model to explain the Euclidean distance between a pair of rule clusters in function of the number of rules in between or the number of trials in between, a p-value for the contribution of the predictor is calculated. The table shows the p-values per individual animal, and the entire data set, for the contribution of each of the two possible predictors of the model: number of rules (first column) and the trials in between (second column).

Paths	# Neurons	Percentage
**South – East**	90	30
**South – West**	84	28
**North – East**	87	29
**North - West**	89	29.67

Supplementary table 2a: Number of neurons significantly correlated to at least one coefficient in each of the four possible paths (Total number of neurons = 300). Using a linear model we explain the firing rates of individual neurons as a function of the trajectory and the speed of the animal (see Methods). We divided the data in the 4 possible paths that the animal could take from a starting arm to a goal arm (South–East, South–West, North–East, North–West). We counted the number of neurons in which at least one of the predictors (three for the trajectory and one for the speed) was significantly contributing to their firing rates (column one). The percentage is calculated by dividing the column one over the total number of neurons (n = 300).

Coefficients:	a	b	c	s
	# Neurons	# Neurons	# Neurons	# Neurons
**South – East**	26	26	25	30
**South – West**	37	33	40	47
**North – East**	34	33	39	47
**North - West**	33	37	49	44

Supplementary table 2b: Number of neurons significantly correlated to at least one coefficient in each of the four possible paths. Similar to Supplementary table 2a, using a linear model we explain the firing rates of individual neurons as a function of the trajectory and the speed of the animal (see Methods). We divided the data into 4 possible paths that the animal could take from a starting arm to a goal arm (South–East, South–West, North–East, North–West). We counted the number of neurons in which every possible predictor (a,b and c for the trajectory and 's' for the speed) was a significant contributor to the explanation of the firing rate.
